# Choosing the right treatment - combining clinicians’ expert knowledge with data-driven predictions

**DOI:** 10.3389/fpsyt.2024.1422587

**Published:** 2024-09-03

**Authors:** Eduardo Maekawa, Esben Jensen, Pepijn van de Ven, Kim Mathiasen

**Affiliations:** ^1^ Department of Electronic and Computer Engineering, University of Limerick, Limerick, Ireland; ^2^ Health Research Institute, University of Limerick, Limerick, Ireland; ^3^ Research Unit for Digital Psychiatry, Centre for Digital Psychiatry, Mental Health Services of Southern Denmark, Odense, Denmark; ^4^ Department for Psychology, Faculty of Health Sciences, University of Southern Denmark, Odense, Denmark; ^5^ Department of Clinical Research, Faculty of Health Sciences, University of Southern Denmark, Odense, Denmark

**Keywords:** Bayesian network, machine learning, mental disorders, digital psychiatry, clinician

## Abstract

**Context:**

This study proposes a Bayesian network model to aid mental health specialists making data-driven decisions on suitable treatments. The aim is to create a probabilistic machine learning model to assist psychologists in selecting the most suitable treatment for individuals for four potential mental disorders: Depression, Panic Disorder, Social Phobia, or Specific Phobia.

**Methods:**

This study utilized a dataset from 1,094 individuals in Denmark containing socio-demographic details and mental health information. A Bayesian network was initially employed in a purely data-driven approach and was later refined with expert knowledge, referred to as a hybrid model. The model outputted probabilities for each disorder, with the highest probability indicating the most suitable disorder for treatment.

**Results:**

By incorporating expert knowledge, the model demonstrated enhanced performance compared to a strictly data-driven approach. Specifically, it achieved an AUC score of 0.85 vs 0.80 on the test data. Furthermore, we evaluated some cases where the predictions of the model did not match the actual treatment. The symptom questionnaires indicated that these participants likely had comorbid disorders, with the actual treatment being proposed by the model with the second highest probability.

**Conclusions:**

In 90.1% of cases, the hybrid model ranked the actual disorder treated as either the highest (67.3%) or second-highest (22.8%) on the test data. This emphasizes that instead of suggesting a single disorder to be treated, the model can offer the probabilities for multiple disorders. This allows individuals seeking treatment or their therapists to incorporate this information as an additional data-driven factor when collectively deciding on which treatment to prioritize.

## 1 Introduction

Identifying the right treatment for mental disorders often requires assessments performed by trained professionals, making the intake time-consuming and costly. Furthermore, it is crucial to consider how to deliver these treatments in a scalable manner to ensure they reach those in need efficiently.

Data-driven models for referral to treatment are of particular interest because it has long been observed that clinicians tend to place more trust in their intuition than is justified by the available evidence (1). However, even the most accurate estimates from experts can result in unsatisfactory outcomes. For instance, several sophisticated and complex psychotherapy studies were conducted to identify subtypes of patients who responded better to alcoholism treatment. Unfortunately, none of these approaches succeeded (2).

Therefore, the implementation of a data-driven referral system for matching treatments with patients can be beneficial for both clinicians and patients. The utilization of machine learning (ML) provides psychologists with a robust framework for making decisions faster based on empirical data rather than relying solely on subjective assessments.

Traditionally, ML models have been designed and employed with the purpose of predicting individual mental disorders one at a time (3–5). This approach, while effective, has limitations in capturing the complexity of mental health conditions that are characterized by overlapping symptoms. Analyzing one disorder at a time overlooks the opportunity to examine comorbid disorders. This makes it difficult to determine which disorder is more likely given the overlapping symptoms (6).

A few studies have used ML to predict mental disorders in multiclass classification problems (7–9). However, many of these lack explainability (10, 11), rendering it challenging for humans to comprehend the recommendations. Some attempts have been made to use more explainable techniques, but they often neglect probabilistic approaches (12–14), which are beneficial for handling uncertainty. One approach to addressing both issues in one model is by utilizing Bayesian networks (BN).

A BN is a graph-based technique that visually represents the interactions among variables, facilitating a clear understanding of their mutual impacts. The use of BNs permits the calculation of conditional probabilities, allowing the model to update its posterior probabilities based on new evidence (15). Due to their probabilistic nature, BNs by default allow the user to obtain quantitative information on the uncertainty associated with predictions. This ability to quantify uncertainty is invaluable in the context of mental health diagnoses, as it enhances the explainability of the model.

The existing literature does not provide a comprehensive understanding of how applying explainable probabilistic machine learning models for multiclass classification scenarios of mental disorders addresses uncertainty and can enhance clinical practice. Our proposed method aims to address these gaps. This study explores probabilistic machine learning to aid mental health specialists in making informed decisions on suitable treatments, offering an objective path to decision-making.

The goal is to create an explainable probabilistic machine learning model to assist therapists in selecting the most appropriate treatment for individuals experiencing Depression, Panic Disorder, Social Phobia, or Specific Phobia.

## 2 Material and methods

### 2.1 Data

The data was gathered from patients, who applied to Internetpsykiatrien for treatment between November 14^th^ 2019 and December 31^st^ 2022. Internetpsykiatrien is a routine care iCBT clinic with nationwide coverage in Denmark. This internet-delivered health care service is funded publicly and is free of charge at the point of use (16). The data comprises individuals over 18 years old, based in Denmark. It includes information about sociodemographic factors such as age, gender, civil status, number of children, education, and income, as well as health questionnaires including the Patient Health Questionnaire (PHQ-9), Panic Disorder Severity Scale-Self Report (PDSS-SR), Generalized Anxiety Disorder (GAD-7), Social Interaction Anxiety Scale (SIAS), and Fear Questionnaire (FQ). These variables serve as inputs for developing the model. All input data is self-reported by patients upon application. Additionally, the data contains our outcome of interest: the “Treatment” prescribed by the clinic for: Depression, Panic Disorder, Social Phobia, and Specific Phobia. In order to determine which treatment to prescribe, a licensed psychologist or a psychologist under supervision of a licensed psychologist conducts a one-hour video assessment with the patient using the Mini International Neuropsychiatric Interview (M.I.N.I.) (17).

**Table A2** in the **Supplementary Material** provides a summary of the characteristics of the socio-demographic variables.

### 2.2 Data processing

We partitioned the data with 1,094 observations into training and testing sets. The split was stratified by “Treatment”, ensuring that each treatment category was represented proportionally in both sets. Specifically, 85% of the data was allocated for training the model, while the remaining 15% was reserved for testing its performance. To avoid any bias, all investigations to determine the categorization of variables were carried out exclusively on the training data. When it was time to evaluate the performance of the model on unseen data, we replicated the same data processing steps on the test data.

Initially, we filtered only the individuals who completed the PDSS-SR, SIAS, and FQ questionnaires. When applying for treatment, patients indicate whether they seek treatment for 1) anxiety, 2) depression, 3) anxiety and depression, or 4) they do not know. If an individual only seeks treatment for depression, they only receive the PHQ-9 and GAD-7 at application. It is unlikely that these individuals will receive an anxiety treatment and hence the utility of a machine learning model that recommends treatment for such individuals is low. Therefore, we focused on cases with all five questionnaires were completed.

For the SIAS questionnaire, it was necessary to reverse the order of the values for items 5, 9, and 11. This adjustment was made because these specific questions follow the opposite direction in terms of severity scale compared to the rest of the items. Additionally, we de-constructed the FQ questionnaire into “MAINPHOBIA”, “TOTALPHOBIA”, “AGORAPHOBIA”, “BLOODPHOBIA”, “SOCIALPHOBIA”, “GLOBALPHOBIA”, and “FQANXIETY”, following the methodology outlined in (18).

Subsequently, we calculated the sum of the values for the original questionnaires and the de-constructed ones, and then we discretized them into groups (SIASgroup, PHQgroup, PDSSgroup, FQgroup, GADgroup, MAINPHOBIAgroup, TOTALPHOBIAgroup, AGORAPHOBIAgroup, BLOODPHOBIAgroup, SOCIALPHOBIAgroup, GLOBALPHOBIAgroup, FQANXIETYgroup).

In the data-driven approach, we used the original range values for the socio-demographic variables “Gender,” “Civil Status,” “Education,” and “Income” from the gathered data. For “Age” and “Number of Children,” we categorized the values as illustrated in **Table 1**. For PHQgroup, GADgroup, and PDSSgroup, we utilized predefined groups from the literature (19–21), respectively. For the remaining variables, we aggregated them by “Treatment”, calculated their averages, and defined the groups based on these averages to differentiate the treatments for each disorder. The resulting aggregated table is presented as **Table A1** (**Supplementary Material**).

**Table 1:** Range groups of data driven and hybrid variables.

Variable	Data driven range of the groups	Hybrid range of the groups
Age	18-21, 22-25, 26-35, 36-44, 46-55, 56-65, ≥66	18-21, 22-25, 26-35, 36-44, 46-55, 56-65, ≥66
Gender	"Female", "Male"	"Female", "Male"
Civil Status	"Single", "Relationship and live together", "Relationship but live alone"	"Single", "Relationship and live together", "Relationship but live alone"
Number of Children	0, 1, 2, 3, ≥4	0, 1, 2, 3, ≥4
Education	"Primary school", "High school", "Vocational Education", "Short education (≤3 years)", "Intermediate education (4 or 5 years)", "Long education (≥5 years)", "Other"	"Primary school", "High school", "Vocational Education", "Short education (≤3 years)", "Intermediate education (4 or 5 years)", "Long education (≥5 years)", "Other"
Income	"Employed", "Social security", "Sickness/benefit pay", "Unemployment benefit", "Stipendium", "Other"	"Employed", "Social security", "Sickness/benefit pay", "Unemployment benefit", "Stipendium", "Other"
PHQgroup	0-4, 5-9, 10-14, 15-19, ≥20	0-9, 10-19, ≥20
GADgroup	0-4, 5-9, 10-14, ≥15	0-9, 10-14, ≥15
FQgroup	0-40, 41-50, 51-60, ≥61	0-50, 51-100, ≥100
PDSSgroup	0-2, 3-7, 8-10, 11-15, 16-18, ≥19	0-10, 11-19, ≥20
SIASgroup	0-21, 22-30, 31-40, ≥41	0-15, 16-40, ≥41
MAINPHOBIAgroup	0-2, 3-5, ≥6	0-2, 3-4, ≥5
TOTALPHOBIAgroup	0-20, 21-30, ≥31	0-20, 21-40, ≥41
AGORAPHOBIAgroup	0-5, 6-10, ≥11	0-9, 10-19, ≥20
BLOODPHOBIAgroup	0-5, 6-10, ≥11	0-10, 11-15, ≥16
SOCIALPHOBIAgroup	0-10, 11-14, 15-20, ≥21	0-9, 10-20, ≥21
GLOBALPHOBIAgroup	0-2, 3-5, ≥6	0-2, 3-5, ≥6
FQANXIETYgroup	0-10, 11-20, ≥21	0-10, 11-20, ≥21
HasPhobia	0 - 1	0 - 1
Treatment	"Depression", "Panic disorder", "Social phobia", Specific phobia"	"Depression", "Panic disorder", "Social phobia", Specific phobia"

In the hybrid method approach, we used the same range groups for the socio-demographic variables. For the remaining variables, we categorized them into three groups, as outlined in **Table 1**. The decision to use only three levels was made in consensus with the expert, as fewer groups provide more data within each group.

### 2.3 Bayesian network model

A Bayesian Network (BN) is a directed acyclic graph consisting of nodes and edges and is used to create an approximation of the joint probability distribution over all variables and the outcome of interest. In this graph, nodes represent variables, while directed edges, depicted by arrows, illustrate relationships among these variables (22). For each node X of the BN it is possible to identify its parents (nodes with arrows pointing towards X), children (nodes receiving arrows from X), and spouses (nodes with arrows leading to children of X but not directly linked to X).

The joint probability distribution of a Bayesian network is the product of the conditional probability distributions for each node $X_{i}$ given its parents, as represented by Equation 1:


(1) $$P\left(X_{1},\;\ldots ,\;X_{n}\right)=\prod_{i=1}^{n}P(X_{i}|Pa\left(X_{i}\right))$$

where $Pa\left(X_{i}\right)$ denotes the parent of node $X_{i}$ . The conditional probability for a node without parents is simply its prior probability. These probabilities can be derived from data, from domain-specific literature, or through the knowledge of human experts.

A joint probability distribution provides all the information required to make probabilistic inferences on one variable if the other variables in the distribution are known. This distribution satisfies the d-separation property (23) indicating that a node is independent of its non-descendants given its parents. Consequently, another important property of a BN is the Markov blanket of a node X, denoted as MB(X). The MB(X) consists of the parents, children, and spouses of node X. The MB is important because it encapsulates the full set of variables influencing or influenced by the node X.

BNs have been successfully applied in various scenarios, including feature selection (24, 25), model prediction (26, 27), and supporting decision-making by providing insights (28–30). Additionally, BNs serve as valuable tools for visualizing and interpreting complex relationships between features, facilitating the identification of key features and their impact on outcomes.

BNs can be constructed using three approaches. The first approach involves utilizing expert knowledge to manually define the connections between nodes. The second approach relies solely on a data-driven approach to learn the BN from the available data. The third approach combines data-driven methods with expert knowledge and is commonly referred to as a hybrid method (31). In this study, we employed two approaches: a purely data-driven approach and a hybrid method.

In the data-driven approach, we constructed the BN using the Expectation-Maximization (EM) algorithm (32) which is advantageous for handling missing values in the data. Specifically, we employed the hill climbing score-based structure learning method (33) with the Bayesian Information Criterion (BIC) score. The hill climb search is a greedy search algorithm that seeks to maximize a score, commencing from an initial random starting point. Subsequently, the algorithm iteratively attempts to identify the set of nodes and connections that maximizes the score.

The hillclimb algorithm is utilized within the structural expectation-maximization (EM) algorithm. Specifically, the EM algorithm commences with an initial random parameter estimation (values of the conditional probability distribution). These parameters are then used to calculate the posterior distributions of the nodes given their parents and to complete missing values using the outputs of the posterior distribution. Subsequently, with this new version of the complete data, a Bayesian Network is constructed using the hillclimb algorithm to maximize the BIC score and re-estimate the parameters. This iterative process continues until the parameters converge.

To enhance the likelihood of obtaining the best Bayesian network, we implemented 1000 random restarts and introduced 5 perturbations for each restart. Perturbations involve additional attempts to refine the Bayesian network structure by introducing, removing, or reversing connections.

In the hybrid approach, based on the data-driven approach, the BN was constructed by incorporating existing knowledge, where the expert added the connections between nodes manually and defined directions based on their expertise. In instances where the expert’s certainty regarding connections or directions was lacking, we conducted data-driven tests to explore all possible scenarios of this uncertainty by adding, removing, and reversing connections. Subsequently, we selected the BN that exhibited superior performance based on evaluation metric Area Under the receiver operating Characteristic curve (AUC). The second author (EKJ) provided the expert knowledge to adapt the network based on experience working in the clinic. EKJ is a psychologist with experience from the clinic, and is now working as a PhD fellow.

Once the Bayesian network was defined for both approaches, we utilized the training data to compute the conditional probability distributions (CPD) associated with each node. This process, known as parameter learning involves applying the Bayesian method (34). Next, predictions for the response variable “Treatment” were made using exact inference (15), wherein the posterior probability is calculated based on a set of events. These events consist of all possible values of the nodes contained within the Markov blanket of the “Treatment” node.

To develop the Bayesian Network model, we utilized the bnlearn package in R (35).

### 2.4 Model evaluation and comparison

To evaluate the data-driven and hybrid models in a statistically robust fashion, we employed repeated cross-validation with 7-folds, repeated 20 times using different random samples from the training data. In each of the 20 iterations, the dataset was divided into subsets, allowing the models to be trained on 6-folds and tested on 1-fold. For each fold, we recorded the F1-score and AUC (36, 37).

The AUC measures the model’s ability to distinguish between classes. The F1-score, on the other hand, is the harmonic mean of the proportion of true positive predictions among all positive predictions and the proportion of true positive predictions among all actual positive instances. Both metrics range from 0 to 1, where higher values indicate better performance.

The final evaluation is performed using the average of these two metrics across all 140 iterations for each disorder, thus providing a statistically robust estimate of the model’s performance. Additionally, we will compute a weighted unique AUC and weighted unique F1-score for the model.

Following the training and validation on the training data, we utilized the remaining 15% portion of the data for a final evaluation of the performance of both approaches on yet unseen data.

### 2.5 Bayesian credible intervals

To quantify the uncertainty in the model outcomes, Bayesian credible intervals are used to provide a confidence level around the predictions. These intervals are calculated using the High Posterior Density (HPD) interval, which includes the most probable values given the observed data and prior information, ensuring the total probability within the interval equals a specified level (e.g., 95%). Among intervals with the same coverage probability, the HPD interval is the shortest, meaning it contains parameter values with the highest posterior density, offering the most precise representation of the credible region (38).

To calculate the Bayesian credible intervals, a nonparametric bootstrap with 100 samples was generated. Using the BN defined in the hybrid approach, the CPDs were learned for each of these samples as discussed in Section 2.3. This process enables obtaining the posterior distribution for each test data observation using the exact inference method described in the same section. Finally, the 95% High Posterior Density (HPD) intervals were calculated along with the probability predictions for each mental disorder.

### 2.6 Comparison with machine learning models

In this study, we compared the Bayesian network model with two other models: Naive Bayes (39) and a Rule-based model. To ensure a fair comparison, we used identical splits of training and testing data for all three models and compared their AUC and F1-score metrics. The methods for developing and evaluating the models are described in Sections 2 and 3 of the **Supplementary Material**.

## 3 Results

### 3.1 Processing missing data

From the initial 1,094 cases, the split provided 932 cases for training and 162 cases for testing. As described in section 2.2, to avoid any data snooping, all the analysis was conducted on the training data until it was necessary to evaluate the model on new, unseen data.

After the data processing described in section 2.2, there were 2.2% missing values in the training data, concentrated in the ‘TOTALPHOBIAgroup’, ‘FQANSIETYgroup’, and ‘CivilStatus’ variables. Initially, we used the data with missing values while employing the EM algorithm described in section 2.3.

Later, these missing values were filled using the “parents” imputation method, performed by the EM algorithm. This method uses the conditional probability distribution of the parent nodes to fill in the missing values.

### 3.2 Bayesian network topology

#### 3.2.1 Development of the data driven network

**Figure 1:**
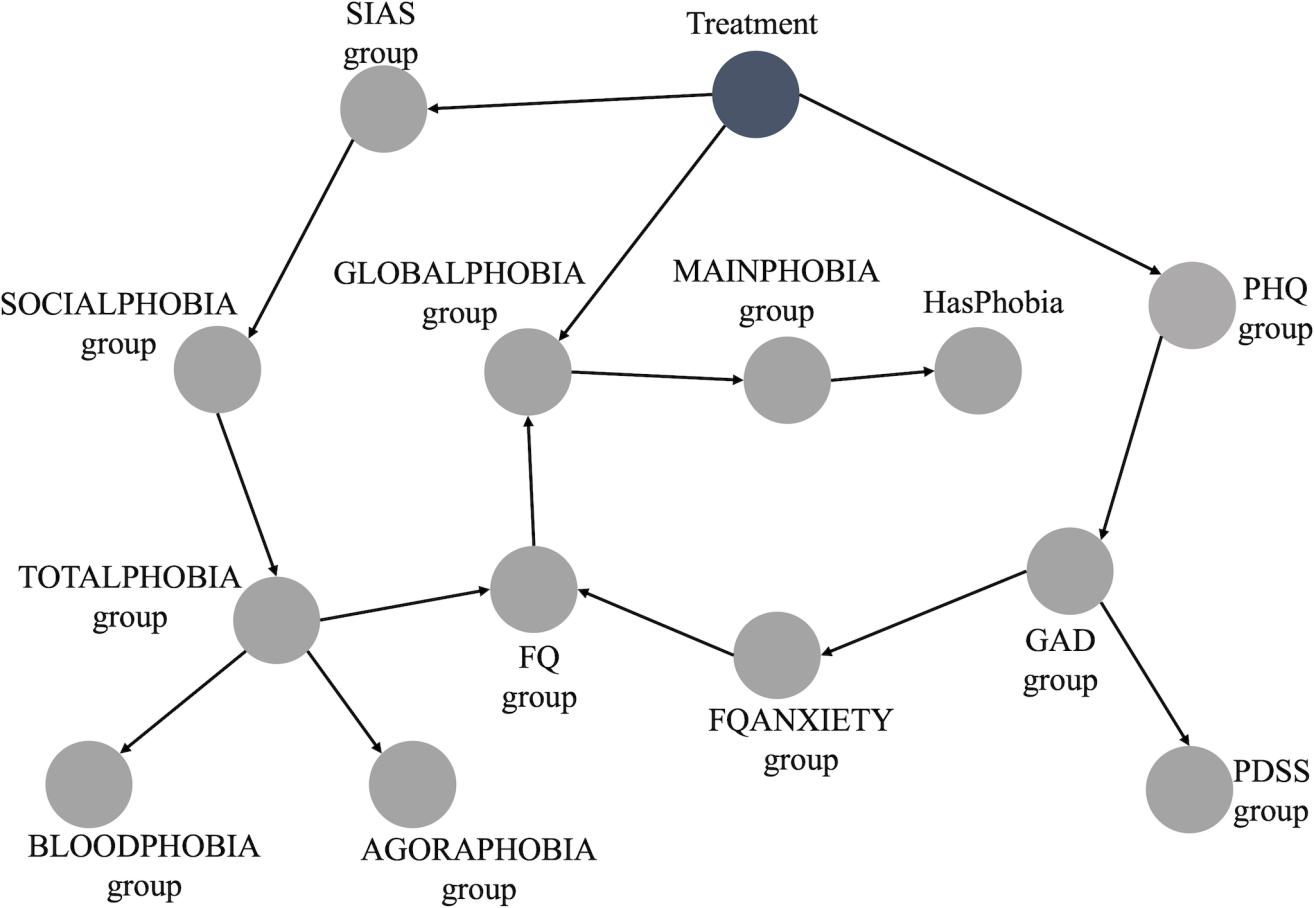
Bayesian network built from data.

The BN constructed from the data is depicted in **Figure 1**. This BN is deemed the optimal representation of the joint probability distribution of our entire dataset using our data-driven approach. From the initial 20 variables used to build the BN (**Table 1**), we retained 14. All socio-demographic variables were discarded as they did not hold significance within the network. These variables were not connected in any way to the main network presented in **Figure 1**, indicating they were completely independent of any node in the main BN, including the outcome of interest, “Treatment”.

Furthermore, it suffices to analyze only the Markov Blanket of the node of interest (“Treatment”) when making predictions through exact inference (40). This holds, because for inference, all nodes apart from the Treatment node can be assumed to contain known information. And in case all nodes in the Markov Blanket are known, any other nodes do not provide extra information. In this scenario, the Markov Blanket consists of the following nodes: “SIASgroup,” “PHQgroup,” “GLOBALPHOBIAgroup,” and “FQgroup” as described in the methods section. The ranges for “SIASgroup,” “PHQgroup,” “GLOBALPHOBIAgroup,” and “FQgroup” are 0-80, 0-27, 0-8, and 0-216, respectively. **Table 1** displays the range groups for each of these nodes.

#### 3.2.2 Adding expert knowledge to the network

**Figure 2:**
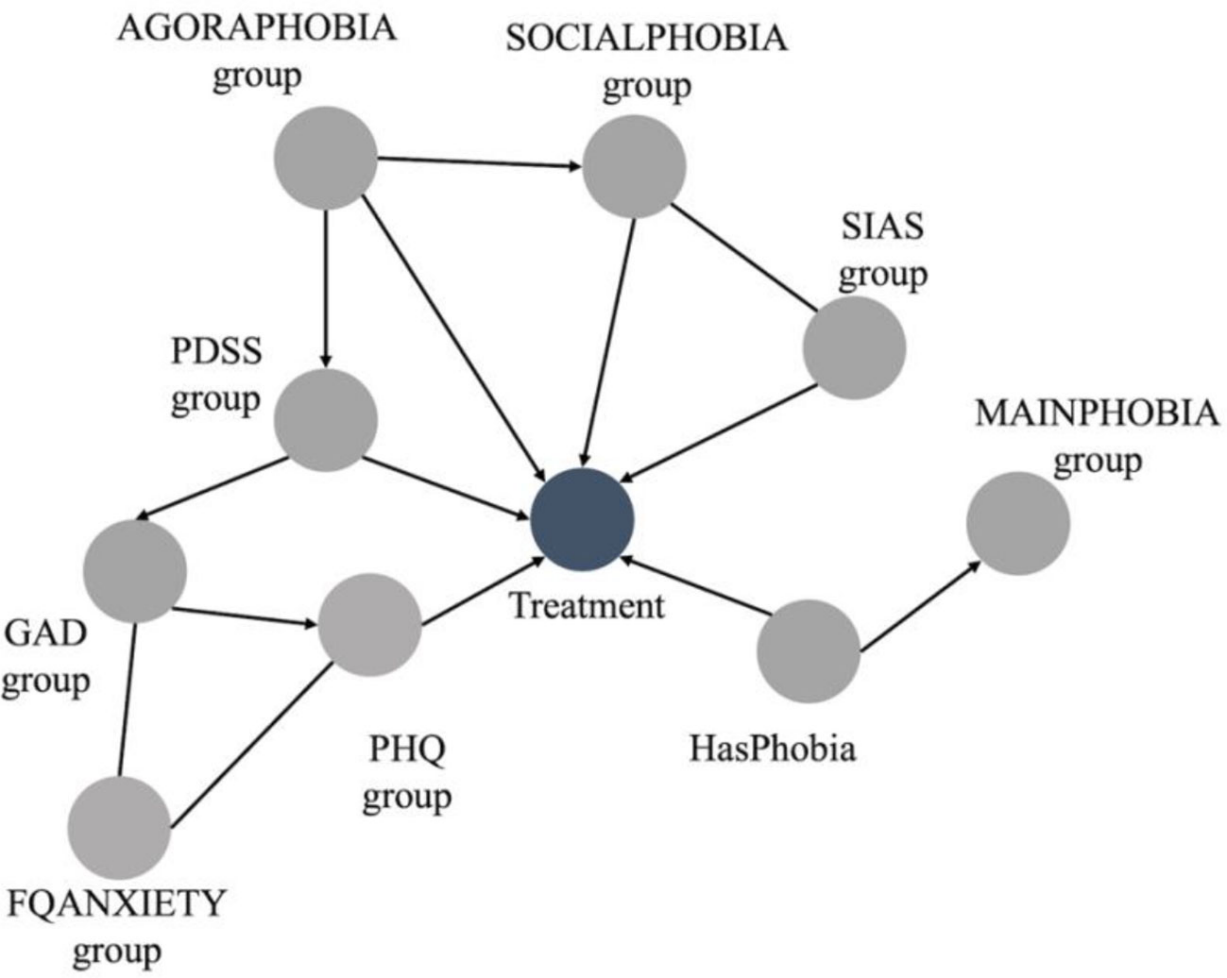
Bayesian network combining data-driven approach and expert knowledge.

**Figure 2** illustrates the BN incorporating the expert knowledge. In this case, “FQgroup” was discarded because it is the summation of “MAINPHOBIAgroup,” “AGORAPHOBIAgroup,” “BLOODPHOBIAgroup,” “SOCIALPHOBIAgroup,” “GLOBALPHOBIAgroup,” and “FQANXIETYgroup.” Similarly, “TOTALPHOBIAgroup” was discarded since it is the summation of “AGORAPHOBIAgroup,” “BLOODPHOBIAgroup,” and “SOCIALPHOBIAgroup”.

“GLOBALPHOBIAgroup” and “BLOODPHOBIAgroup” were also absent from the BN. This omission, made by the expert, was deliberate. The global phobia subscale is a generic questionnaire without any diagnostic indications. The severity of other questionnaires and FQ subscales were considered to have more diagnostic value.

The blood-phobia subscale covers a range of specific phobias that would all be treated in the specific phobias program. Given that the treatment program focuses on the treatment of one phobia, the diagnostic value lies in identifying a dominant phobia rather than aggregating multiple phobias into one group. Therefore, the “HasPhobia” item and the “MAINPHOBIAgroup” subscales were considered to be better indicators.

Furthermore, some items of the blood-phobia subscale could be confounded by the presence of other disorders. For instance, hospitals could be an anxiety-provoking situation for agoraphobic individuals as their distance from home may be far and hospital visits usually include exposure to large groups of people. For individuals with social phobia they may provoke anxiety because a visit likely involves talking to strangers.

In summary, the described changes made based on the expert’s input resulted in a BN with 10 variables. From the 14 variables selected in the data-driven, the 4 health-related variables (“GLOBALPHOBIAgroup,” “BLOODPHOBIAgroup,” “FQgroup,” and “TOTALPHOBIAgroup”) were discarded as discussed previously.

##### 3.2.2.1 Uncertainties in the expert knowledge

**Figure 2** shows some connections without direction. Specifically, these connections are between “SOCIALPHOBIAgroup” and “SIASgroup”, between “FQANXIETYgroup” and “PHQgroup”, and between “FQANXIETY” and “GADgroup.” These connections represent the expert’s uncertainty regarding their directions.

#### 3.2.3 Finalizing the hybrid approach

Regarding the undirected connections, since they do not affect the Markov Blanket of the outcome, we chose to discard them. This decision also resulted in the exclusion of the node “FQANXIETYgroup”.

Furthermore, there are overlapping symptoms between the nodes “AGORAPHOBIAgroup” and “SOCIALPHOBIAgroup”. Thus, to ensure we have the best BN structure for our model, we explored various BN structure scenarios: a) keeping only the connection from “AGORAPHOBIAgroup” to “Treatment”; b) keeping only the connection from “SOCIALPHOBIAgroup” to “Treatment”; c) discarding both connections; d) retaining connections from both nodes to “Treatment”.

For each scenario, we evaluated the performance metrics discussed in section 2.3 and ultimately selected the structure that demonstrated the best performance. The combination with the superior performance involved discarding both connections: the connection from “AGORAPHOBIAgroup” to “Treatment” and the connection from “SOCIALPHOBIAgroup” to “Treatment”.

**Figure 3:**
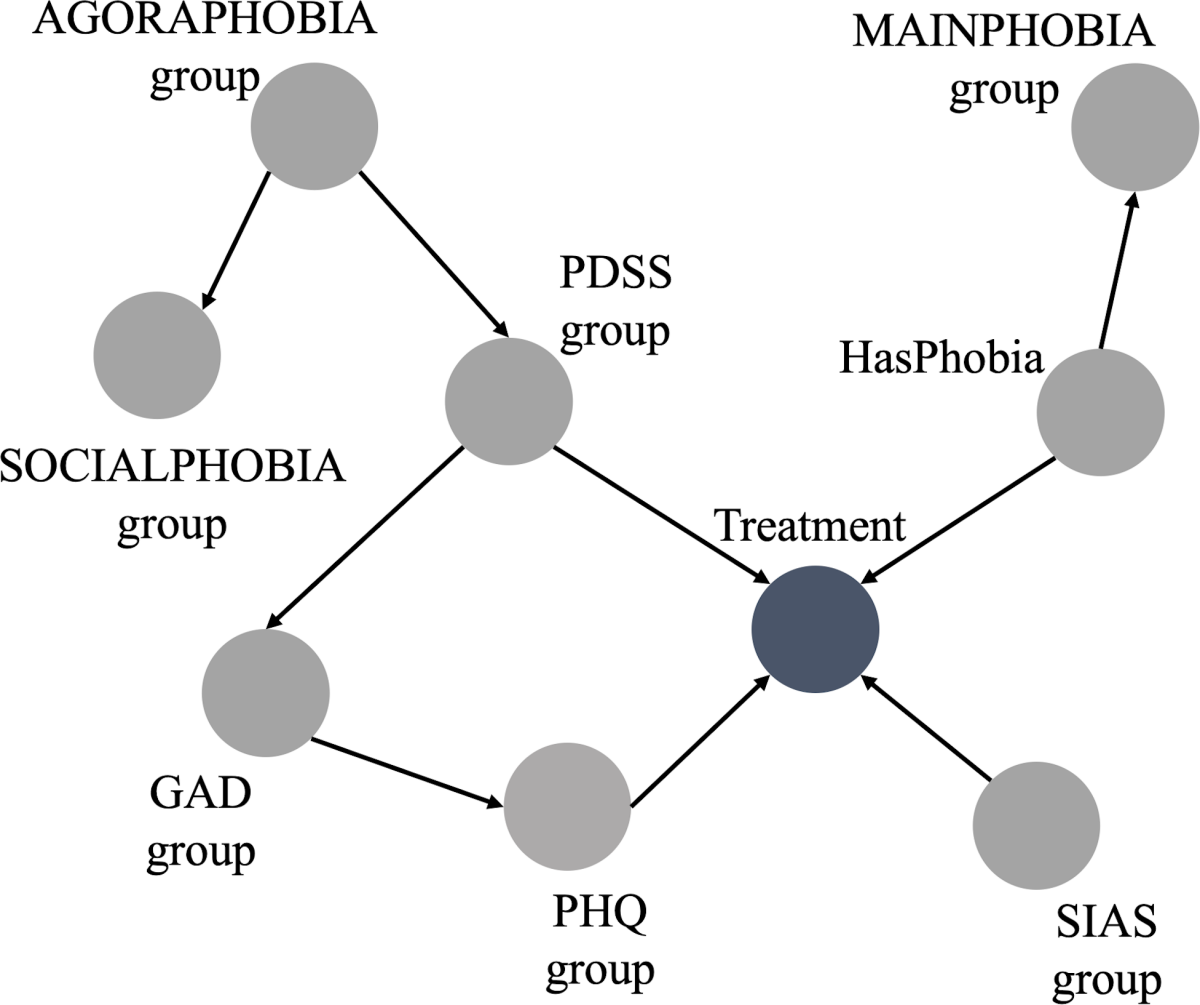
Bayesian network using hybrid approach.

The BN derived from the hybrid approach is displayed in **Figure 3**. The range groups for the nodes in this Bayesian network are listed in **Table 1**, as discussed in section 2.2.

The hybrid BN now consists of 9 variables, derived from the expert BN inputs which has 10 variables, with the exclusion of “FQANXIETYgroup”.

### 3.3 Evaluation of the model

The performance of both approaches is summarised in **Table A3** (**Supplementary Material**) which lists the average repeated cross-validation F1 and AUC score. For each disorder, **Table A3** (**Supplementary Material**) also presents the minimum, maximum values and the standard deviation of these metrics across all the iterations. This provides insight into the variation of metrics when using different subsets of the training set.

Comparing the results in **Table A3** (**Supplementary Material**) for the Data-Driven and Hybrid model shows that employing the hybrid approach resulted in improved metrics across all disorders to be treated. To obtain a single metric for the AUC and the F1-score across the four disorders, values were weighted by the prior distributions of each class. The total F1-scores were 0.59 and 0.67, while the total AUC values were 0.79 and 0.86 for the data-driven and hybrid approaches, respectively. This suggests that the hybrid approach is likely to outperform the solely data-driven approach when applied to new data. However, to confirm this assertion, it is essential to utilize new, unseen data to validate whether the results from the cross-validations hold.

**Table A4** (**Supplementary Material**) displays the results on the test data, comprising the remaining 15% of the data that was initially split at the outset of model development. Once more, all metrics in the hybrid approach surpassed those of the data-driven approach. Moreover, all metrics in the test data fall within the range of the minimum and maximum values observed in the repeated cross-validation table.

The weighted average F1-scores over the four disorders were 0.57 and 0.66, while the weighted average AUC values were 0.80 and 0.85 for the data-driven and hybrid approaches, respectively. The AUC of 0.85 in a multi-class classification problem with four classes (and hence a chance level of 0.25) is promising, surpassing a similar study that employed deep learning techniques to also address a multi-label classification problem for mental disorders (41), where the overall weighted AUC was 0.79.

### 3.4 Comparison of BN model with naïve Bayes and rule-based model

For the Naive bayes model, all categorical variables were transformed into ordinal numbers. Subsequently, we addressed missing data, which accounted for 2.2% of the dataset, by assigning them to a new category, as outlined in (42).

The use of RFECV for feature selection resulted in the retention of 17 variables including the outcome. Specifically, we excluded ‘TOTALPHOBIAgroup’, ‘Education’, and ‘Income’ from the original set of 20 variables as shown in **Table 1**.

For the rule-based model, there was no need to fill in missing values since the variables selected, as discussed in section 2.6, were already complete.

The results of these models, presented in **Table A5** (**Supplementary Material**), include AUC and F1 values. For the Naive Bayes model these were obtained through cross-validation with 140 evaluations. As the Rule-based model does not contain trainable parameters, **Table A5** (**Supplementary Material**) shows results from a single evaluation of the training data. The AUC and F1 values were calculated by weighting each metric of each disorder according to its prior distribution, as discussed in sections 2.4.

Comparing the BN model with Naive Bayes, **Table A5** (**Supplementary Material**) indicates that they perform similarly in terms of AUC, but the BN model outperforms Naive Bayes in terms of F1 score. The Rule-based model shows slightly worse AUC and underperforms in terms of F1 score.

In **Table A6** (**Supplementary Material**), results from a single evaluation on the test data are presented. Naive Bayes performs slightly better in terms of AUC compared to the BN model but slightly worse in F1 score. The Rule-based model shows a slightly lower AUC and underperforms in terms of F1 score, which is in conformance with the model’s performance on training data.

## 4 Discussion

### 4.1 Model output

The output of the model provides four probabilities corresponding to each mental disorder to be treated. Additionally, it includes the important variables and their values, as shown in **Table 2**. These significant variables constitute the Markov blanket of our outcome of interest, and they are sufficient for making predictions using exact inference.

**Table 2:** Output of the BN model for 4 salient cases as represented by their interview scores.

Case	PHQgroup	PDSSgroup	HasPhobia	SIASgroup	Depression	Panic Disorder	Social Phobia	Specific Phobia
1	0-9	0-10	1	≥41	9.12%	0.04%	90.80%	0.04%
2	≥20	0-10	0	21-40	94.37%	0.03%	5.58%	0.03%
3	10-19	0-10	0	0-20	82.47%	12.51%	5.01%	0.01%
4	0-9	11-19	0	21-40	0.02%	67.97%	24.00%	8.01%

PHQgroup,: group range that contains total score for PHQ.

PDSSgroup: group range that contains total score for PDSS.

HasPhobia: presence (1) or absence (0) of phobia.

SIASgroup: group range that contains total score for SIAS.

Depression: probability for depression treatment.

Panic Disorder: probability for panic disorder treatment.

Social Phobia: probability for social phobia treatment.

Specific Phobia: probability for specific phobia treatment.

In the 4 cases presented in **Table 2** the model correctly predicted that individual 1 received Social Phobia treatment, while cases 2 and 3 received depression treatment, and case 4 received treatment for Panic Disorder. By modelling the probabilistic relationships between variables, it becomes possible to trace the influence of different factors on the outcome, thereby enhancing the explainability of the model’s predictions. Furthermore, the Bayesian credible intervals (discussed in more detail in section 4.3) provide a method to quantify uncertainty by integrating prior knowledge and updating information based on observed data via Bayes’ theorem. This enables the representation and propagation of uncertainty throughout the model, resulting in more reliable and interpretable outcomes.

### 4.2 Rankings of the model

**Figure 4:**
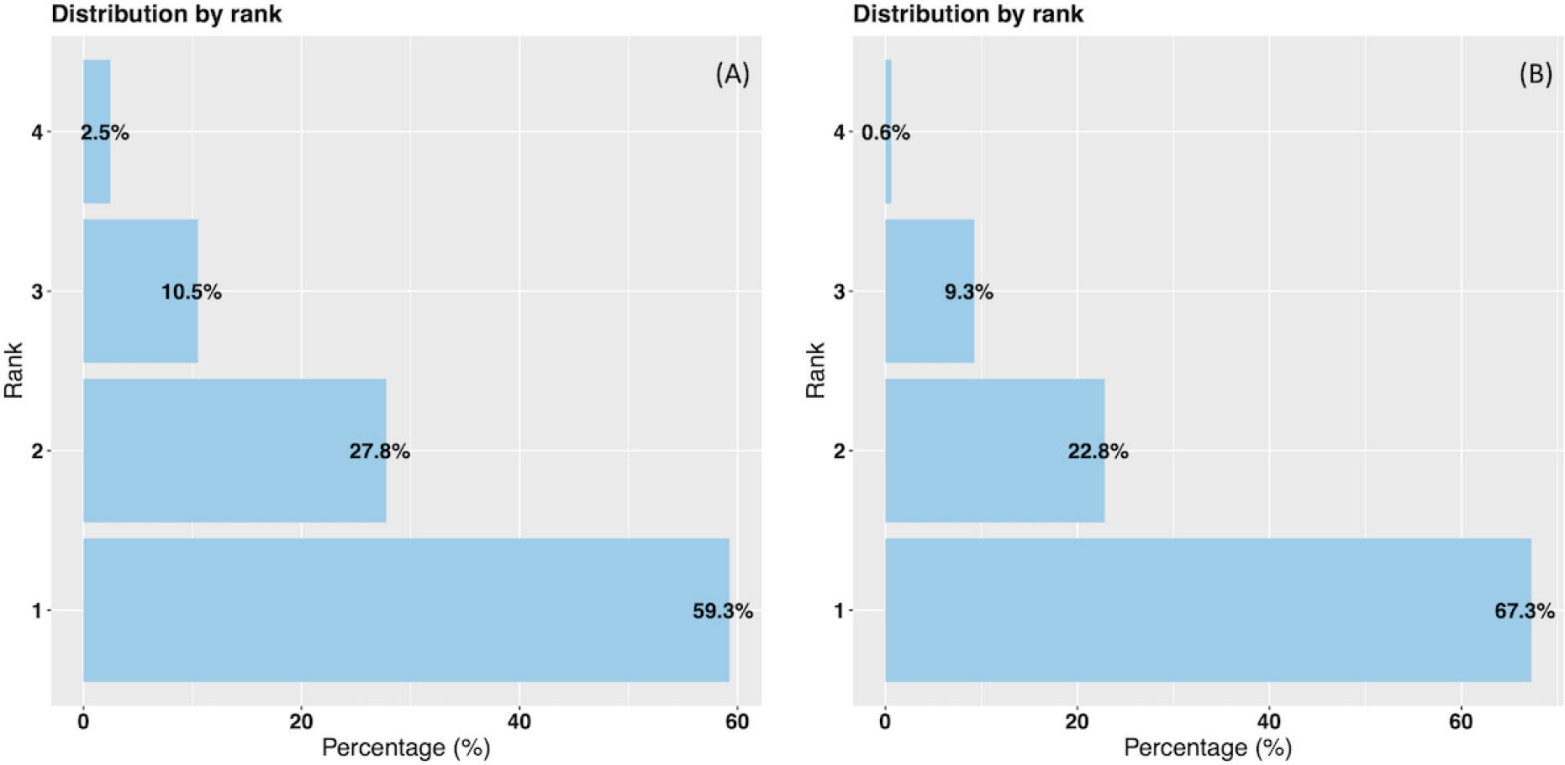
Data-driven and hybrid predictions ranked probabilities vs % of actual disorder treated. **(A)** Data-driven approach. **(B)** Hybrid approach.

The probabilities of the model are ranked from highest to lowest, with each probability corresponding to a specific disorder to be treated. In **Figure 4A**, we observe the distribution of these probabilities based on ranks using the data-driven approach on the test data. Rank 1 indicates that the highest probability predicts the actual disorder treated, while rank 2 indicates that the second highest probability corresponds to the actual disorder treated, and so forth for ranks 3 and 4. Thus, in 59.3% of cases, the model allocated the highest probability to the actual disorder treated. The second, third and fourth probabilities matched the actual disorder treated in 27.8%, 10.5% and 2.5% of instances, respectively.

In **Figure 4B** representing the hybrid approach, we also observe improved results on the test data. Specifically, in 67.3% of instances, the highest probability class matched the actual class. The second, third and fourth probabilities corresponded to the actual disorder treated in 22.8%, 9.3% and 0.6% of instances, respectively. Notably, in 90.1% of cases, either the highest or the second highest probability aligns with the correct disorder treated.

To examine any patterns in misclassification, we examined how many patients were misclassified for each treatment in the test data. The total numbers can be seen in **Table 3**.

**Table 3:** Number of correctly classified and misclassified cases for each disorder.

	Actual Treatment			
Model Prediction	Depression	Panic Disorder	Social Phobia	Specific Phobia
Depression	34	6	5	2
Panic Disorder	8	42	8	6
Social Phobia	8	6	28	2
Specific Phobia	0	2	0	5

For depression, the model was equally likely to misclassify the treatment as panic disorder or social phobia. For panic disorder, a similar pattern was seen with equal probability of being misclassified as either depression or social phobia. Social phobia was misclassified as panic disorder slightly more often than depression. For all three disorders, the model never or very rarely misclassified the treatment as specific phobia. For specific phobias, the proportion of misclassifications was higher, which is not surprising given the small number of data samples for this treatment.

The model misclassified 32% of depression treatments, 25% of panic disorder treatments, and 31.7% of social phobia treatments. Given how prevalent co-morbid depression and anxiety are, with rates as high as 58.3% in primary care samples (43), these levels of misclassifications are not surprising. Patients with comorbid disorders will present with elevated scores on both anxiety and depression questionnaires, and the model provides its bests guess, without any social context or medical history to help decide on one treatment over the other.

We decided to take a closer look at some of the cases where the model’s second guess was the actual disorder treated instead of the first guess. We wanted to see, if we could interpret why the model suggested treating a different disorder than the one actually treated for these patients and whether the model’s estimates could still be useful. **Table 4** displays four cases where there is a mismatch between the predictions of the model and the actual disorder treated. Additionally, it presents the range groups of the variables utilized for prediction estimation. The detailed range values can be found in **Table 1**, and the model’s predicted probabilities for each disorder are in **Table 5**.

**Table 4:** Variable values of mismatched cases between predicted and actual disorder treatment.

	Case 5	Case 6	Case 7	Case 8
Model prediction	Depression	Social Phobia	Panic Disorder	Specific Phobia
Disorder treatment	Panic Disorder	Depression	Specific Phobia	Panic Disorder
PHQgroup	≥ 20	10-19	10-19	0-9
PDSSgroup	11-19	0-10	11-19	0-10
HasPhobia	0	0	1	1
SIASgroup	21-40	≥ 41	0-20	0-20

**Table 5:** Probabilities of the model for each disorder treatment.

	Case 5	Case 6	Case 7	Case 8
Depression	74.0%	37.2%	0.0%	0.0%
Panic Disorder	22.2%	4.7%	66.6%	16.2%
Social Phobia	3.7%	55.8%	0.0%	2.7%
Specific Phobia	0.0%	2.3%	33.4%	81.1%

Case 5 and case 6 both illustrate cases where the model was mistaken about depression and anxiety treatment. In case 5, the model might have favored depression treatment due to the relatively high PHQ-9 score, whereas the model might have favored social phobia in case 6 due to the high SIAS score. We can try to understand the clinician’s decision on one treatment over the other in different ways. For example, a clinician might find that symptoms of depression would prevent a patient from working effectively with their anxiety. Therefore, they would focus on depression treatment first for a patient with co-morbid anxiety and depression. On the other hand, the medical history of a patient could indicate that the anxiety disorder caused the depression. Therefore, treating the anxiety disorder could alleviate the depression as well. In both cases, it is plausible that either depression or anxiety treatment could have been helpful. The clinician could decide on what they thought most appropriate, or the patient could decide what to focus on based on which symptoms currently interfere most with their daily life.

For both case 7 and case 8 the model was wrong about panic disorder and specific phobia. In case 7, the model seemed to favor panic disorder over the specific phobia due to a combination of elevated PHQ-9, PDSS, and FQ agoraphobia scores. The symptom presentation seems somewhat complex. Therefore, it is interesting that the clinician focused on the specific phobia. Case 7 might be an example of patient preference being a deciding factor for the treatment decision. Often, individuals seeking treatment for a specific phobia have a clearly defined problem they want help with and are keenly aware of the source of their fear. Case 7 might have preferred this treatment, even if they presented with other symptoms.

In case 8, the model seemed to favor specific phobia due to the relatively low scores on all measures aside from FQ main phobia. Case 8 has the overall lowest symptom scores of the four cases. This type of symptom presentation, with low scores on most questionnaires but high scores on the FQ main phobia question, is characteristic of individuals seeking treatment for specific phobias at the clinic. Thus, it is understandable that the model favored the specific phobia in this case. The model still caught that the FQ agoraphobia subscale was slightly elevated, giving panic disorder treatment second priority.

All four cases show the usefulness of the ranked output of the model. Instead of deciding on one specific treatment, the model can be used to provide the two most likely treatment options. Individuals seeking treatment, or their therapists, can use this information as an extra data-driven factor in deciding on which treatment to focus. Case 7 is a good example of why this joint decision-making could be useful. The model predicted two possible treatments favoring panic disorder based on the symptom presentation, but the individual might be more interested in treating their specific phobia. Possibly because this phobia interfered more with the individual’s life than the panic disorder symptoms.

### 4.3 Prediction analysis

**Figure 5:**
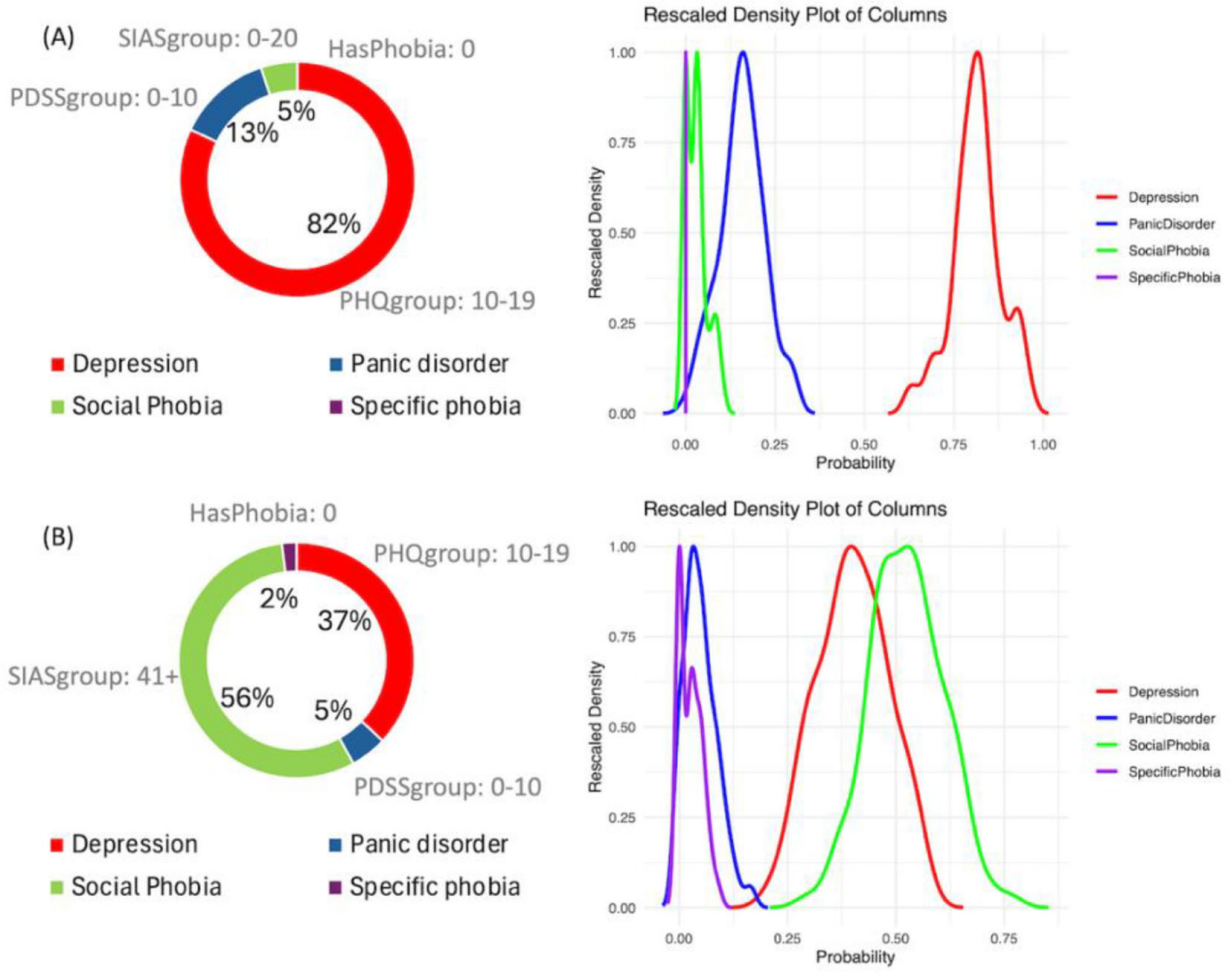
Examples of output of the Bayesian network model. **(A)** Case where model prediction and the disorder treated are correct. **(B)** Case where model predicted Social phobia treatment and the actual treatment was for depression.

We selected two cases from the model outputs to illustrate how the analysis can be conducted. One case demonstrates alignment between the model’s predictions and the actual treatment (**Figure 5A**), while the other case shows a mismatch between the prediction and the treatment (**Figure 5B**) Guidelines for clinicians is also provided in Section 4.4.

**Figure 5A** illustrates case 3 discussed in section 4.1 (**Table 2**), in which the prediction of the model and the disorder treated are aligned. It is evident that the prediction of depression treatment shows an 82% probability, with no overlap in its posterior distributions compared to other disorders. Additionally, it is possible to examine the values of the most important variables that explain the decision: SIASgroup with a score between 0 and 20 (out of 80), PDSSgroup with a score between 0 and 10 (out of 28), PHQgroup with a score between 10 and 19 (out of 27), and the absence of HasPhobia. These four variables compose the Markov blanket of the outcome. The Bayesian credible intervals can be viewed in **Table 6**.

**Table 6:** Bayesian credible intervals - model prediction and disorder treatment correct.

	Depression	Panic disorder	Social phobia	Specific phobia
Credible Interval - min	0.68	0.04	0.001	0.001
Probability	0.82	0.13	0.05	0.001
Credible Interval - max	0.96	0.30	0.09	0.002

**Figure 5B** illustrates case 6 discussed in section 4.2, where the model predicted Social Phobia treatment but the actual treatment was for Depression. It is evident that there is a notable overlap between the distributions of Depression treatment and Social Phobia treatment, with the probability of Social Phobia treatment being 56% compared to 37% for Depression treatment. The probability predictions and the Bayesian credible intervals can be observed in **Table 7**.

**Table 7:** Bayesian credible interval - model prediction Social phobia treatment and the actual treatment was for Depression.

	Depression	Panic disorder	Social phobia	Specific phobia
Credible Interval - min	0.25	0.001	0.36	0.01
Probability	0.37	0.05	0.56	0.02
Credible Interval - max	0.55	0.12	0.68	0.07

Less overlap between the distributions of each mental disorder indicates higher confidence in the prediction. Moreover, overlapping distributions may suggest the presence of more than one disorder.

### 4.4 Model applicability in supporting decision-making

**Figure 6:**
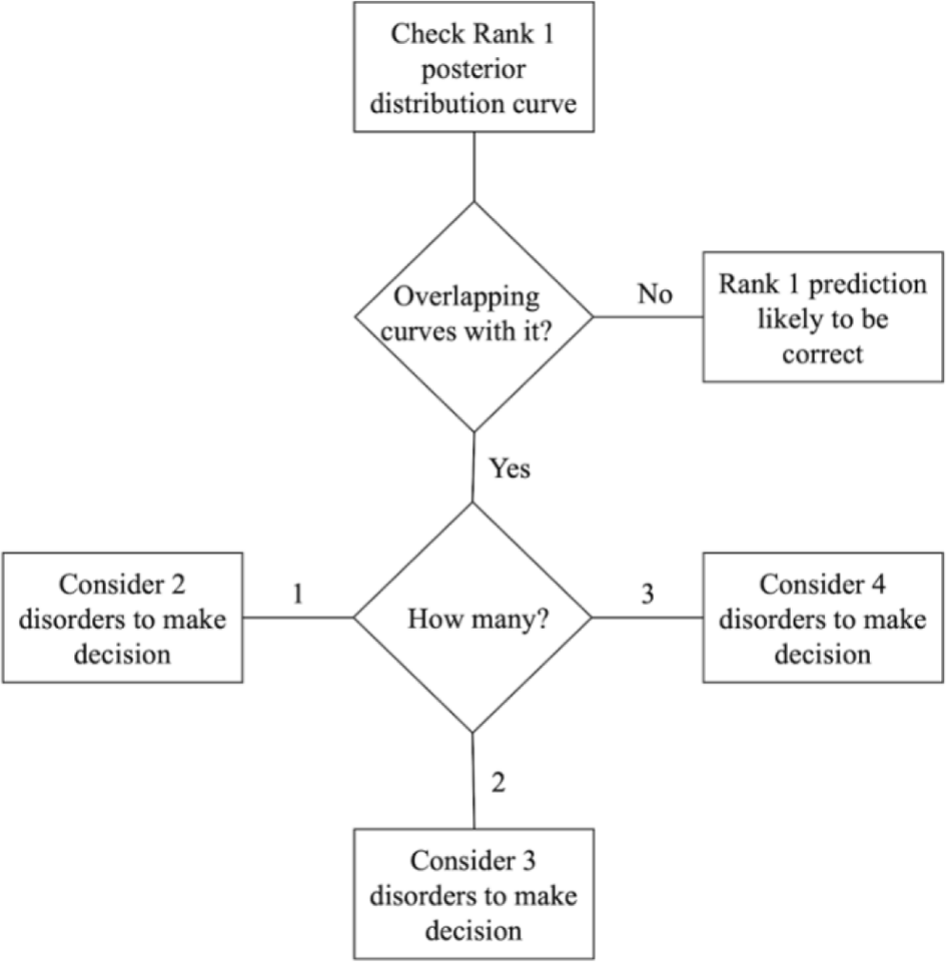
Flowchart explaining how to use the model to aid the decision-making process.

The BN model provides two important outputs that should be analyzed together: the ranked probabilities of having each of the four disorders and the Bayesian credible intervals of the predictions, as shown in **Figure 5A**. This information can be useful for making treatment decisions. **Figure 6** illustrates a step-by-step guide on how to use this information for the final decision, which includes the following four steps:

1. Check the highest probability disorder (Rank 1) as shown in the doughnut chart on the left in **Figure 5A**.
2. Examine the overlapping curves in the posterior distribution chart on the right in **Figure 5A**.
3. If the curve related to the highest probability disorder has no overlap, it is very likely that the Rank 1 prediction is correct.
4. If the Rank 1 curve overlaps with other curves, identify the disorders associated with these overlapping curves. The clinician should then use their knowledge of the mutual influence of these disorders on the treatment options.

The number of overlapping curves indicates which ranked probabilities need to be considered when making a decision for the appropriate treatment. Having more than one overlapping curve can facilitate a discussion with patients about possible diagnoses and treatments, enabling a joint decision-making process that benefits both the clinician and the patient.

### 4.5 Advantages of the Bayesian network model

The results in **Table A5** (**Supplementary Material**) indicate that the Naive Bayes model performs similar to the BN model in terms of predictive accuracy and that both Bayesian models outperform the Rule-based model. Aside from predictive performance, BNs provide some important advantages over the other two models.

Firstly, the BN model achieves comparable performance using only 4 variables, whereas Naive Bayes uses 16 variables and the Rule-based model uses 7 variables. This simplicity makes the BN model less complex and easier to interpret, facilitating explainability of its predictions.

Secondly, the BN model provides additional benefits in terms of explainability compared to the Rule-based model. It offers insights extracted from data through probabilistic measures, rather than relying on predefined rules. This approach can reveal relationships and dependencies between variables that may not be apparent from common clinical knowledge.

Thirdly, although Naive Bayes is a probabilistic model, its assumption of independence between variables limits its ability to explain how certain combinations of symptoms affect decisions compared to other combinations. This independence assumption leads to an overly simplistic view of comorbidity.

## 5 Conclusions

The aim of the study was to propose a methodology using a Machine learning model that uses historic data and human expertise to make sound and interpretable treatment recommendations for four mental disorders an individual will likely need: Depression, Panic Disorder, Social Phobia and Specific Phobia. In this study, we applied 2 different approaches to develop the model. One approach employed a purely data-driven methodology, while the other adopted a hybrid approach, combining knowledge from domain experts with data-driven techniques.

In 90.1% of cases, the hybrid model ranked the actual disorder treated the highest (67.3%) or second-highest (22.8%). Furthermore, the output of the model provides information on the most important variables and can indicate when to consider one, two, three, or all four ranked probabilities for making an appropriate treatment decision. This highlights the capacity of the model to serve as a valuable tool in prioritizing disorders for treatment, benefiting both individuals seeking treatment and their therapists. This approach has the potential to facilitate joint decision-making based on data-driven information.

The performance of the model with expert knowledge appears promising, suggesting that it could be effectively utilized in real-world scenarios. By modelling uncertainty, facilitating explainability, and incorporating domain expertise, our approach offers valuable tools for building reliable and transparent diagnostic systems.

We compared the Bayesian network model with two other models, Naive Bayes and a Rule-based model. The performance of the BN and Naive Bayes model were similar and both outperformed the Rule-based model. The BN model offers the advantage of being less complex compared to the other two models, which aids explainability.

### 5.1 Limitations and perspectives

The size of the training data used is not large enough to fully mitigate potential biases. For instance, because we developed a model to predict four different classes, the limited amount of data can lead to heightened sensitivity to outliers, particularly in the minority classes. This sensitivity can affect the model’s performance and generalization, especially if the minority classes are underrepresented in the training data.

While iCBT has been proven to be an effective treatment capable of scaling up and has the prospect of being a cost-effective delivery format of CBT with a large reach, triage of patients is still a barrier. It is costly to perform assessment interviews, which need to be performed by highly skilled personnel. Furthermore, most assessments lead to non-eligibility. It is therefore of significant benefit to efficient use of available resources, to be able to automate parts of the screening process. The present analysis shows promising results in this regard, thus prompting for additional research in this area. Additionally, the methodology described in this study could be applied at item level for the questionnaires to examine which items are most important for diagnostic purposes. Thus, it might be possible to shorten the questionnaires, and reduce the burden of completing several screening instruments for people applying for treatment.

## Data Availability

The data analyzed in this study is subject to the following licenses/restrictions: Data cannot be made publicly available, due to the sensitive nature of routine care clinical data. Requests to access these datasets should be directed to esjensen@rsyd.dk.
