# Supplementary material for: Choosing the right treatment - combining clinicians’ expert knowledge with data-driven predictions
Source: Front Psychiatry. 2024 Sep 3;15:1422587. doi: 10.3389/fpsyt.2024.1422587 (PMC11406075; doi:10.3389/fpsyt.2024.1422587)
Supplement: Supplementary file 1 [file Table1.docx]

Supplementary Material

Choosing the right treatment - Combining Clinicians’ Expert Knowledge with data-driven predictions

Eduardo Maekawa^1,2*^, Esben Jensen^3,4^, Pepijn van de Ven Maekawa^1,2^, Kim Mathiasen^,3,5^

^1^ Department of Electronic and Computer Engineering, University of Limerick, Limerick V94 T9PX, Ireland

^2^ Health Research Institute, University of Limerick, Limerick, Ireland

^3^ Research Unit for Digital Psychiatry, Centre for Digital Psychiatry, Mental Health Services of Southern Denmark, Odense, Denmark

^4^Department for Psychology, Faculty of Health Sciences, University of Southern Denmark, Odense C, Denmark

^5^Department of Clinical Research, Faculty of Health Sciences, University of Southern Denmark, Odense C, Denmark

*** Correspondence:**Eduardo Maekawa
Eduardo.maekawa@ul.ie

# Supplementary Tables

Table A1. Mean of total scores of variables by Treatment

| Treatment | Depression | Panic Disorder | Social Phobia | Specific Phobia |
| --- | --- | --- | --- | --- |
| PHQT | 17.79 | 11.27 | 11.68 | 6.87 |
| GADT | 13.12 | 12.48 | 11.17 | 7.26 |
| PDSST | 9.39 | 14.63 | 9.55 | 9.66 |
| FQT | 54.69 | 57.67 | 64.30 | 45.22 |
| SIAST | 34.91 | 26.05 | 44.96 | 18.31 |
| MAINPHOBIA | 0.96 | 1.74 | 2.43 | 5.83 |
| TOTALPHOBIA | 26.71 | 31.70 | 37.01 | 17.81 |
| AGORAPHOBIA | 7.50 | 11.98 | 9.77 | 4.82 |
| BLOODPHOBIA | 5.57 | 9.06 | 6.15 | 6.82 |
| SOCIALPHOBIA | 13.63 | 10.66 | 21.09 | 6.17 |
| GLOBALPHOBIA | 2.63 | 3.94 | 4.56 | 5.67 |
| FQANXIETY | 22.26 | 17.87 | 17.54 | 12.50 |
| HasPhobia | 0.17 | 0.30 | 0.46 | 0.87 |

Table A2. Table of characteristics

| Variable | Description | N | % |
| --- | --- | --- | --- |
|  |  |  |  |
| Treatment | Depression | 336 | 30.7% |
|  | Panic Disorder | 377 | 34.5% |
|  | Social Phobia | 276 | 25.2% |
|  | Specific Phobia | 105 | 9.6% |
|  |  |  |  |
| Age | 18-21 | 127 | 11.6% |
|  | 22-30 | 243 | 22.2% |
|  | 31-40 | 387 | 35.4% |
|  | 41-50 | 161 | 14.7% |
|  | 51-60 | 104 | 9.5% |
|  | 61 or over | 50 | 4.6% |
|  |  |  |  |
| Gender | Female | 329 | 30.1% |
|  | Male | 765 | 69.9% |
|  |  |  |  |
| CivilStatus | Single | 395 | 36.1% |
|  | Relationship and live together | 550 | 50.3% |
|  | Relationship, but live alone | 149 | 13.6% |
|  |  |  |  |
| NumberChildren | 0 | 689 | 63.0% |
|  | 1 | 116 | 10.6% |
|  | 2 | 205 | 18.7% |
|  | 3 | 71 | 6.5% |
|  | 4 or more | 13 | 1.2% |
|  |  |  |  |
| Education | Primary school | 111 | 10.1% |
|  | High school | 266 | 24.3% |
|  | Vocational Education | 129 | 11.8% |
|  | Short education (<= 3 years) | 95 | 8.7% |
|  | Intermediate education (4 or 5 years) | 271 | 24.8% |
|  | Long education (>= 5 years) | 189 | 17.3% |
|  | Other | 33 | 3.0% |
|  |  |  |  |
| Income | Employed | 506 | 46.3% |
|  | Social security | 8 | 0.7% |
|  | Sickness/benefit pay | 11 | 1.0% |
|  | Unemployment benefit | 72 | 6.6% |
|  | Stipendium | 318 | 29.1% |
|  | Pension | 35 | 3.2% |
|  | Other | 144 | 13.2% |

Table A3. Evaluation of the models. Mean CV is the average of 7 folds cross validation during training. Min-Max is the minimum and maximum of the 7 folds. SD is the standard deviation.

|  | Data-driven | | | Hybrid | | |
| --- | --- | --- | --- | --- | --- | --- |
|  | Mean CV | Min-Max | SD | Mean CV | Min-Max | SD |
| F1-score Depression | 0.62 | 0.51-0.77 | 0.05 | 0.68 | 0.53-0.80 | 0.06 |
| F1-score Panic Disorder | 0.54 | 0.40-0.67 | 0.05 | 0.68 | 0.56-0.81 | 0.05 |
| F1-score Social Phobia | 0.59 | 0.44-0.73 | 0.06 | 0.63 | 0.49-0.78 | 0.05 |
| F1-score Specific Phobia | 0.52 | 0.27-0.80 | 0.11 | 0.60 | 0.35-0.87 | 0.10 |
| AUC Depression | 0.84 | 0.77-0.93 | 0.03 | 0.86 | 0.77-0.91 | 0.03 |
| AUC Panic Disorder | 0.72 | 0.59-0.82 | 0.04 | 0.83 | 0.72-0.90 | 0.04 |
| AUC Social Phobia | 0.84 | 0.72-0.92 | 0.04 | 0.84 | 0.75-0.94 | 0.04 |
| AUC Specific Phobia | 0.88 | 0.72-0.98 | 0.05 | 0.88 | 0.69-0.98 | 0.06 |

Table A4. Performance of the model on test data

|  | Data-driven | Hybrid |
| --- | --- | --- |
| F1-score Depression | 0.64 | 0.70 |
| F1-score Panic Disorder | 0.60 | 0.70 |
| F1-score Social Phobia | 0.61 | 0.66 |
| F1-score Specific Phobia | 0.32 | 0.45 |
| AUC Depression | 0.82 | 0.87 |
| AUC Panic Disorder | 0.70 | 0.83 |
| AUC Social Phobia | 0.84 | 0.86 |
| AUC Specific Phobia | 0.86 | 0.93 |

Table A5. Comparison of performance in training data

| Training | | | |
| --- | --- | --- | --- |
|  | Rule-based | Naive Bayes | Bayesian network |
| AUC | 0.83 | 0.86 | 0.86 |
| F1 | 0.50 | 0.56 | 0.67 |

Table A6. Comparison of performance on test data

| Test | | | |
| --- | --- | --- | --- |
|  | Rule-based | Naive Bayes | Bayesian network |
| AUC | 0.83 | 0.87 | 0.85 |
| F1 | 0.54 | 0.62 | 0.66 |

# Naive bayes

Naive Bayes is a specific type of Bayesian network where the response variable is the parent of each predictor variable, and each predictor has only one directed edge coming from the response. The “naive” aspect refers to the assumption that all predictor variables are independent given the response.

To select the features for developing the model, we used the Recursive Feature Elimination with Cross-Validation (RFECV) approach (1). This process employs a supervised learning estimator that provides feature importance information and iteratively removes the least important variables from the data, while evaluating the model's performance using cross-validation. In this study, we used Naive Bayes as the estimator and the performance metric evaluated in this process was accuracy.

When developing a model, it is common practice to perform hyperparameter optimization, a process of fine-tuning parameters aimed at enhancing the model's performance. To effectively fine-tune these parameters and objectively evaluate model performance, we implemented nested cross-validation (2). Nested cross-validation operates in two steps: an outer and an inner loop.

During the outer loop, the dataset is split into multiple folds. One fold serves as the test set, while the remaining folds constitute the training set. The primary purpose of the outer loop is to evaluate the model's performance.

Nested within the outer loop, the inner loop focuses on hyperparameter tuning. Within this loop, the training set is further partitioned into folds, with one fold reserved as a validation set and the others used for training. Different models, each with different parameters, are trained and evaluated in this process to determine the optimal set of hyperparameters. The optimal set of hyperparameters is typically determined by maximizing a defined metric, such as AUC or F1-score.

Upon completing the inner loop, the best-performing hyperparameters, as determined by their performance on the training folds, are applied to the test dataset reserved in the outer loop. The performance metrics from each iteration of the outer loop are aggregated to provide an overall evaluation of the model's performance.

In this study, we employed nested cross-validation with 7 folds for both the inner and outer loops, repeated 20 times, resulting in 140 evaluations. This approach mirrors the repeated cross-validation utilized in the Bayesian network model. For hyperparameter tuning, we utilized Bayesian optimization to maximize the AUC metric (3) .

The Naive bayes model development was implemented in Python 3.7.7 using MultinomialNB  from sklearn, and BayesSearchCV from skopt. Python is freely available open source software (Python Software Foundation).

# Rule-based model

The rule-based model uses the normalized scores of the variables to predict a treatment. This is done by averaging the normalized scores of all variables associated with each of the disorders of interest. The model then selects disorder/treatment associated with the highest normalized score.

First, we considered all eight variables from the hybrid approach, but instead of using group categories as outlined in Table 1 from main paper, we used the total sum of the original questionnaires as discussed in section 2.2. Then we divided the total score of each variable by its maximum range value, resulting in a value between 0 and 1.

We then grouped the variables into four categories corresponding with the four treated disorders: "Depression", "Panic Disorder", "Social Phobia", and "Specific Phobia”. Specifically, we grouped the variables “SIAS" and “SOCIALPHOBIA" under the “Social Phobia” category. We grouped “AGORAPHOBIA” and “PDSS,” as “Panic Disorder”. For “Specific Phobia,” we grouped “MAINPHOBIA” and “HasPhobia”. Finally, for “Depression,” we used only “PHQ”. We decided to exclude the “GAD” variable, as it indicates generalized anxiety, which was not part of the categories in this study.

For each of the categories, the model averages the normalized scores as described above and decides on a disorder by picking the category with the highest average score.

1. Shahana T, Lavanya V, Bhat AR. Ensemble classifiers for bankruptcy prediction using SMOTE and RFECV. International Journal of Enterprise Network Management. 2024;15(1):109-32.

2. Abdulaal MJ, Casson AJ, Gaydecki P, editors. Performance of Nested vs. Non-Nested SVM Cross-Validation Methods in Visual BCI: Validation Study. 2018 26th European Signal Processing Conference (EUSIPCO); 2018 3-7 Sept. 2018.

3. Victoria AH, Maragatham G. Automatic tuning of hyperparameters using Bayesian optimization. Evolving Systems. 2021;12(1):217-23.
